# Retained stone retrieval basket causing chronic pancreatitis: a case report

**DOI:** 10.3389/fsurg.2023.1235833

**Published:** 2023-08-09

**Authors:** James Wai Kit Lee, Ming Yuan Tan, Calvin Koh, Shridhar Ganpathi Iyer, Yujia Gao

**Affiliations:** ^1^ Department of Surgery, National University Hospital, Singapore, Singapore; ^2^ Department of Surgery, National University of Singapore, Singapore, Singapore; ^3^ Department of Surgery, Alexandra Hospital, Singapore, Singapore; ^4^ Department of Surgery, Khoo Teck Puat Hospital, Singapore, Singapore; ^5^ Department of Gastroenterology, National University of Singapore, Singapore, Singapore

**Keywords:** pancreatitis, retained foreign body, biliary disease, unusual presentation, hepatobiliary surgery, ERCP (cholangiopancreatography)

## Abstract

**Background:**

Endoscopic retrograde cholangiopancreatography is a common procedure performed for choledocholithiasis and gallstone pancreatitis. Although a relatively low risk procedure, it is not without its complications. Cases of impacted Dormia baskets during stone retrieval have been reported, but these are usually retrieved surgically during the same setting.

**Case summary:**

A 40-year-old man presented to our hospital with an episode of epigastric pain and discomfort. He has a prior background of recurrent episodes of pancreatitis of which he underwent prior endoscopic therapy in his home country. Initial investigations revealed a metallic object seen on abdominal x-ray, computer tomographic scan of the abdomen and pelvis, and magnetic resonance imaging of the pancreas. Further evaluation was done with endoscopy, which revealed a retained stone extraction basket from a previous endoscopic retrograde pancreatography, resulting in recurrent episodes of acute chronic pancreatitis. Although the retained foreign body was removed, he subsequently developed further complications of portal vein thrombosis as a result of recurrent acute chronic pancreatitis, which required anticoagulation.

**Conclusion:**

This case highlights the importance of retrieving any foreign body from the pancreas, especially on the head, to prevent the development of further complications.

## Introduction

Up to 20% of patients with symptomatic gallstone disease present with concomitant choledocholithiasis (1). Endoscopic retrograde cholangiopancreatography (ERCP) with stone extraction is a common first-line treatment. Common bile duct (CBD) stones are removed using stone extraction baskets and extraction balloon catheters, with success rates ranging from 80% to 90% (2). The procedure is not without complications (3), and although rare, there have been over 100 documented cases of extraction basket fracture in modern literature (4–8). The common reasons for wire fracture are impaction caused by a hard, large stone, wire fracture due to excessive manipulation, especially with the hand cranking mechanism used for lithotripsy, and inability to close the basket. Because of potential complications such as infection, pancreatitis, migration and bleeding, fractured baskets are usually extracted immediately, either by extending sphincterotomy, balloon dilatation, and shockwave lithotripsy or by laparoscopic exploration (9, 10). In this study, we report the long-term sequalae of a case of a patient with a retained fractured end of the basket on the head of the pancreas, resulting in chronic pancreatitis with gross pancreatic atrophy, pancreatic duct (PD) dilatation, multiple PD stones, and early-onset diabetes mellitus (DM).

## Case presentation

The patient is a 40-year-old male, who was admitted to the emergency department in April 2021 for epigastric pain, which radiated to the back, and vomiting. The patient did not have fever, jaundice, and recent loss of weight or appetite. He was asymptomatic prior to admission. He had a history of gallstone pancreatitis, for which he underwent multiple ERCP procedures overseas between 2008 and 2012. The patient did not have cholecystectomy. He was diagnosed to have DM 3 years ago, which was controlled with oral antidiabetics.

**Figure 1:**
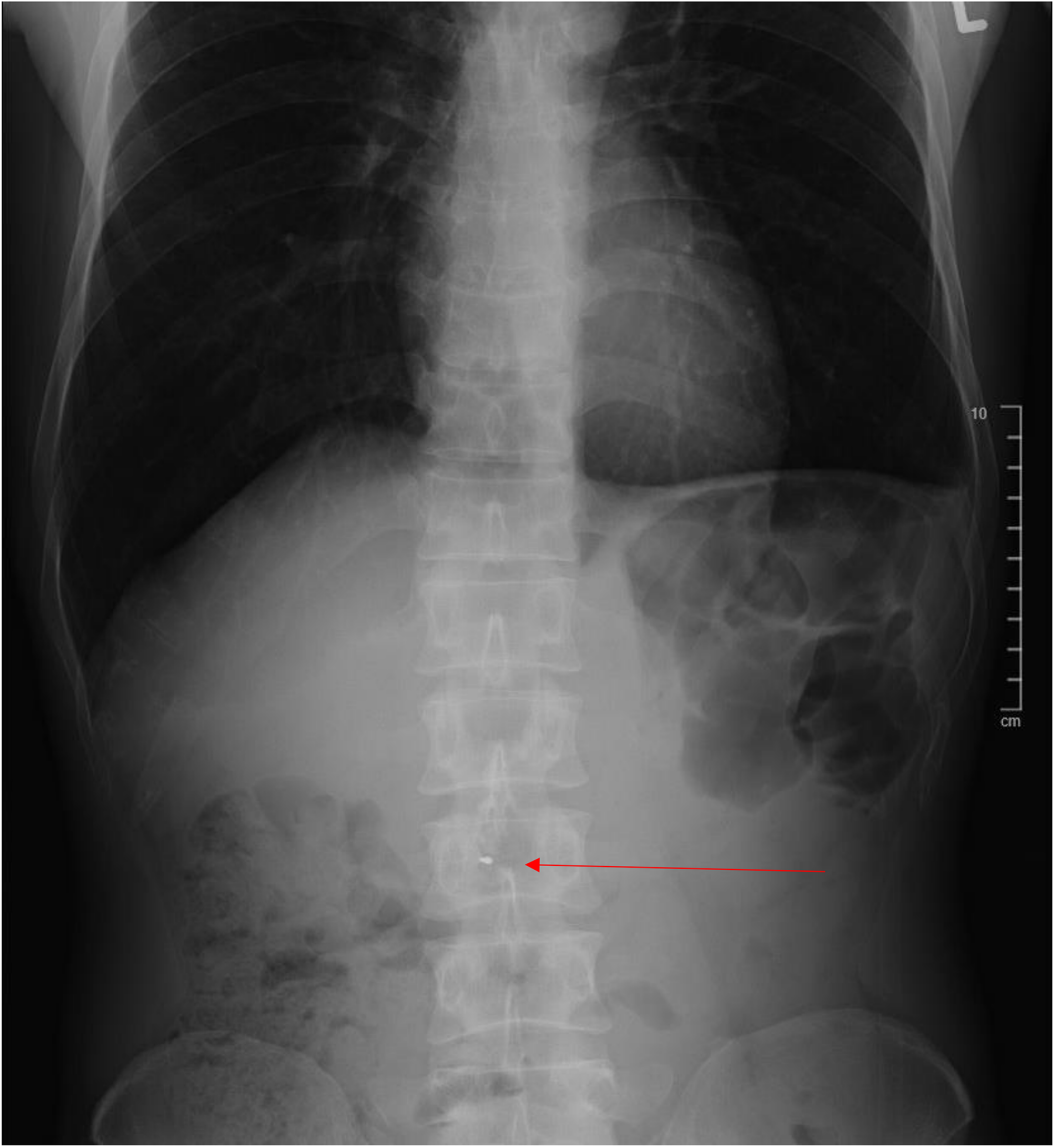
Abdominal x-ray.

**Figure 2:**
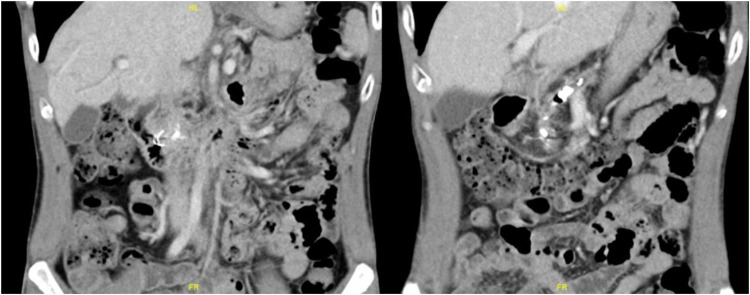
Computer tomographic scan of the abdomen and pelvis (CTAP).

On abdominal examination, it was found that he had epigastric and central abdominal tenderness but no signs of peritonism. Initial investigations showed a white blood cell count of 11.4 × 10^9^/L (normal 4–10 × 10^9^/L), and normal serum amylase and lipase and liver function tests (LFTs) were carried out. Plain abdominal radiograph (Figure 1) showed an abnormal hyperdense object in the central abdominal region. After revisiting the patient’s history, he was informed that a metallic stent had been inserted during his last ERCP. Because of the unusual shape of the object, which did not resemble any known metallic stents, a computed tomography (CT) scan of the abdomen was performed (Figure 2).

The scan showed an unusually shaped hyperdense object on the head of the pancreas, which was distinct from the PD stones seen on the scan. The pancreas was also extremely atrophic with PD dilatation, mild biliary dilatation, and multiple PD calculi throughout the remnant pancreas.

**Figure 3:**
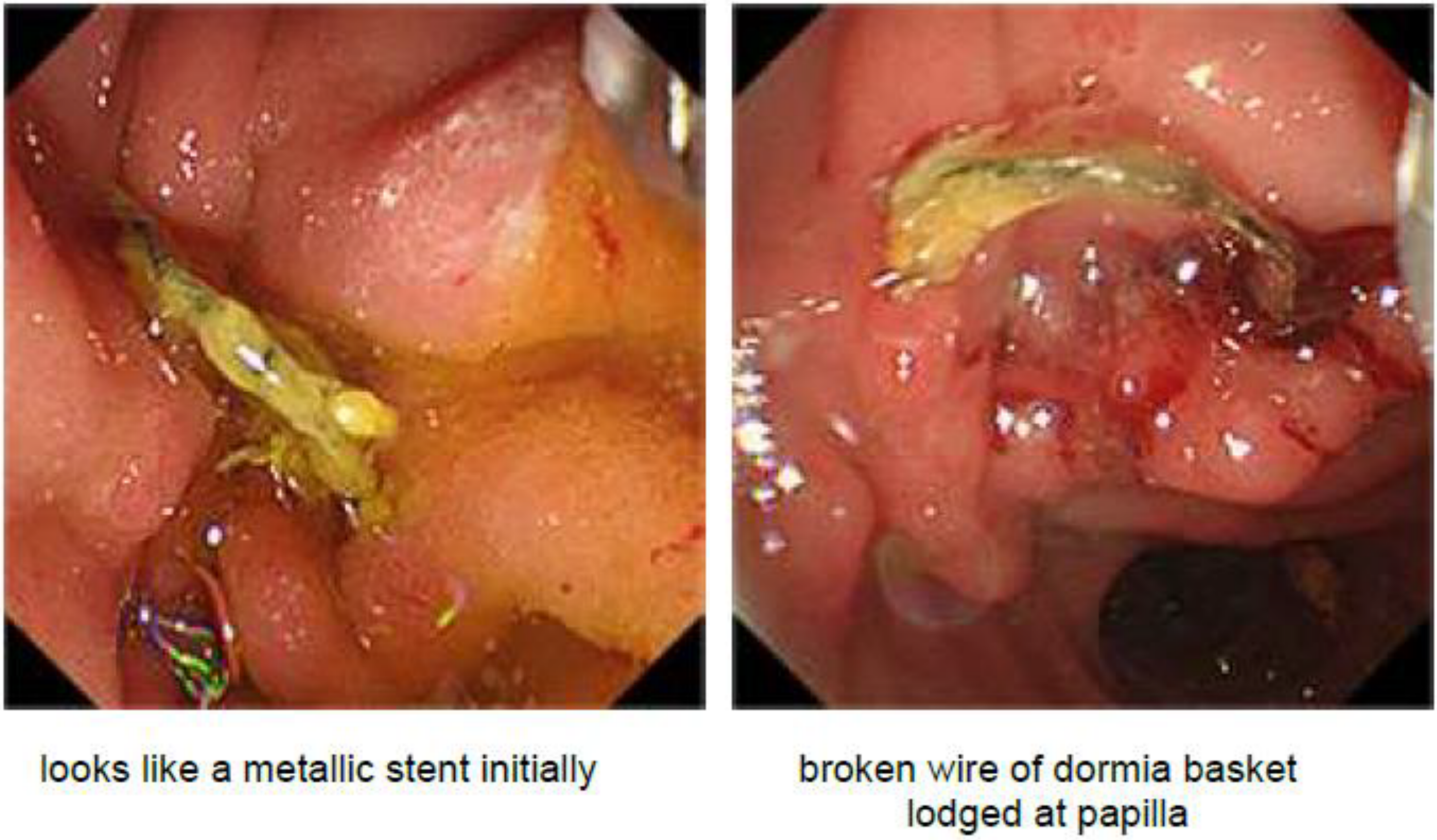
Metallic object on endoscopic evaluation.

**Figure 4:**
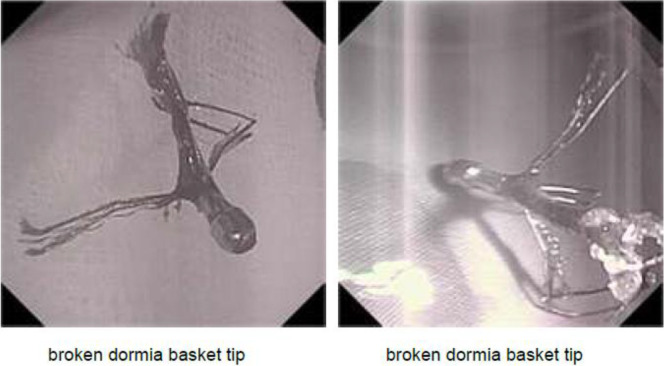
Removal of a retained Dormia basket.

An ERCP was performed, which showed a metallic wire protruding from the ampulla of Vater, which was initially thought to be part of a fractured stent (Figure 3). The foreign body was extracted using an endoscopic grasper. On further examination of the retrieved object, it became clear that it was the fractured end of a Dormia basket, which was likely from the previous ERCP procedure (Figure 4). A balloon was used to remove as many PD stones as possible, but we were unable to clear the PD completely.

The patient recovered well initially and was discharged 2 days after the ERCP. He was readmitted 2 weeks later for central abdominal pain again, and a repeat CT scan showed a thrombosis of the main portal vein with extension into the superior mesenteric vein. The patient was started on anticoagulation with low-molecular-weight heparin and managed conservatively. However, due to worsening symptoms and mesenteric venous edema on subsequent scans with extension of the portal vein thrombus, he underwent a transhepatic catheter–directed portal vein thrombolysis, which resulted in partial success. He was then managed with intravenous heparin infusion with a target APTT ranging between 50 and 60 and subsequently discharged with oral anticoagulation. The patient subsequently sought to return to his home country for the continuation of his treatment, and hence, no long-term information on this condition was available.

## Discussion

Retained foreign bodies are a matter of concern for patient safety. Retained surgical gauze, needles, stents, or other medical instruments have been reported, and they can result in high rates of morbidity and mortality. Despite adequate clinician training, safety checklists, and processes, retained foreign bodies are still reported (11, 12).

Fractured Dormia baskets are an uncommon but reported occurrence during the performance of ERCP. It is estimated that the risk ranges from 1% to 6% (4), with most being caused by impaction with a large stone (more than 15 mm in diameter) or an excessive manipulation of the device, leading to a distortion of the wires by hard calculi. These incidents are usually identified and resolved during the same setting to avoid future complications such as infections, foreign body migration, and pancreatitis among others (11). In a series published by Vargas Ávila et al. (4), it is postulated that a large stone size (more than 15 mm in diameter) seems to be the main risk factor behind Dormia basket impaction, and it tends to occur at the ampulla of Vater just prior to exiting into the duodenum. It is extremely challenging to remove a foreign body from the head of the pancreas, with most case studies reporting balloon dilatation, sphincterotomy extension, and in many cases, open or laparoscopic surgical retrieval. There is no consensus on the best modality to remove impacted Dormia baskets. Mechanical/laser lithotripters or extracoporeal shockwave lithotripsy have been most frequently reported for clearing the debris (7, 8, 13, 14). Boston Scientific’s Trapezoid™ basket is designed to disengage or break at the tip to allow the wires to be retrieved in the event of basket failure. To date, there has been no report in the literature on the long-term effects of a retained foreign body on the head of the pancreas.

In our patient, it is presumed that the fractured Dormia basket was left behind following the last ERCP, which was performed in 2012. No records of the procedure were available, and the patient was only able to recall being informed that a metal stent was placed inside. The retained Dormia basket probably resulted in pancreatic duct dilatation, subclinical chronic pancreatitis, pancreatic ductal stones, atrophy of the pancreas, and diabetes mellitus.

This case highlights the importance of retained foreign bodies in patients. Although an important part of the process is to prevent such events from happening, when they do happen, it is as important for clinicians to have a low threshold to suspect and diagnose a retained foreign body. Then, it becomes imperative that there should be open disclosure to the patient and a discussion about the risks and benefits of removal of the foreign body. In symptomatic patients, understandably, there are more clinical grounds for removal. However in patients who are asymptomatic, one would need to weigh the risks and benefits of retrieval, especially in the context of patients who might be of extremely high risk for such a procedure. All identified retained foreign bodies should be logged in a registry specific to the procedure for documentation and audit. To maintain standards, healthcare institutions should also aim for a Six Sigma Certification for their procedures in line with other industry standards for ensuring defect-free processes and products.

## Conclusion

Dormia basket fracture is a known complication of ERCP procedures, especially when they are used for mechanical lithotripsy. Clinicians may be aware of this, but they must also be aware of the importance of retrieving the fracture fragments, because leaving them behind may lead to dire consequences for the patient. All attempts should be made to retrieve the fractured basket and ensure that no fragments are left behind. It is also imperative that patients be warned of this possibility prior to surgery, as part of the consent-taking process, and full disclosure should also be made should any complications occur during the procedure.

## Data Availability

The original contributions presented in the study are included in the article/Supplementary Material, further inquiries can be directed to the corresponding authors.
